# MSG-15: Super-Bioavailability Itraconazole Versus Conventional Itraconazole in the Treatment of Endemic Mycoses—A Multicenter, Open-Label, Randomized Comparative Trial

**DOI:** 10.1093/ofid/ofae010

**Published:** 2024-01-29

**Authors:** Andrej Spec, George R Thompson, Marisa H Miceli, Justin Hayes, Laurie Proia, David McKinsey, Ana Belen Arauz, Kathleen Mullane, Jo-Ann Young, Gerald McGwin, Rachel McMullen, Tyler Plumley, Mary K Moore, Lee Ann McDowell, Carolynn Jones, Peter G Pappas

**Affiliations:** Division of Infectious Disease, Washington University in St Louis School of Medicine, St Louis, Missouri, USA; Department of Internal Medicine, Division of Infectious Diseases and Department of Medical Microbiology and Immunology, University of California Davis Medical Center, Sacramento, California, USA; Department of Internal Medicine, Division of Infectious Disease, University of Michigan, Ann Arbor, Michigan, USA; Division of Infectious Diseases, University of Arizona College of Medicine, Tucson, Arizona, USA; Department of Medicine, Rochester Regional Health, Rochester, New York, USA; Metro Infectious Disease Consultants, Kansas City, Missouri, USA; Department of Medicine, University of Panama and Hospital Santo Tomas, Panama City, Panama; Department of Medicine/Section of Infectious Diseases and Global Health, University of Chicago, Chicago, Illinois, USA; Department of Medicine, Division of Infectious Disease and International Medicine, Program in Adult Transplant Infectious Disease, University of Minnesota, Minneapolis, Minnesota, USA; Department of Internal Medicine, Division of Infectious Diseases, University of Alabama at Birmingham, Birmingham, Alabama, USA; Department of Internal Medicine, Division of Infectious Diseases, University of Alabama at Birmingham, Birmingham, Alabama, USA; Mycoses Study Group Education and Research Consortium, Birmingham, Alabama, USA; Department of Internal Medicine, Division of Infectious Diseases, University of Alabama at Birmingham, Birmingham, Alabama, USA; Department of Internal Medicine, Division of Infectious Diseases, University of Alabama at Birmingham, Birmingham, Alabama, USA; Mayne Pharma, Medical Affairs, Raleigh, North Carolina, USA; College of Nursing, The Ohio State University College of Nursing, Columbus, Ohio, USA; Mycoses Study Group Education and Research Consortium, Birmingham, Alabama, USA; Department of Internal Medicine, Division of Infectious Diseases, University of Alabama at Birmingham, Birmingham, Alabama, USA; Mycoses Study Group Education and Research Consortium, Birmingham, Alabama, USA

**Keywords:** blastomycosis, coccidioidomycosis, endemic mycoses, histoplasmosis, itraconazole

## Abstract

**Background:**

Invasive fungal disease caused by dimorphic fungi is associated with significant morbidity and mortality. Super-bioavailability itraconazole (SUBA-itra) is a novel antifungal agent with pharmacokinetic advantages over currently available formulations. In this prospective comparative study, we report the outcomes of patients with endemic fungal infections (histoplasmosis, blastomycosis, coccidioidomycosis, and sporotrichosis).

**Methods:**

This open-label randomized trial evaluated the efficacy, safety, and pharmacokinetics SUBA-itra compared with conventional itraconazole (c-itra) treatment for endemic fungal infections. An independent data review committee determined responses on treatment days 42 and 180.

**Results:**

Eighty-eight patients were enrolled for IFD (SUBA-itra, n = 42; c-itra, n = 46) caused by *Histoplasma* (n = 51), *Blastomyces* (n = 18), *Coccidioides* (n = 13), or *Sporothrix* (n = 6). On day 42, clinical success was observed with SUBA-itra and c-itra on day 42 (in 69% and 67%, respectively, and on day 180 (in 60% and 65%). Patients treated with SUBA-itra exhibited less drug-level variability at days 7 (*P* = .03) and 14 (*P* = .06) of randomized treatment. The concentrations of itraconazole and hydroxyitraconazole were comparable between the 2 medications (*P* = .77 and *P* = .80, respectively). There was a trend for fewer adverse events (AEs; 74% vs 87%, respectively; *P* = .18) and serious AEs (10% vs 26%; *P* = .06) in the SUBA-itra–treated patients than in those receiving c-itra. Serious treatment-emergent AEs were less common in SUBA-itra–treated patients (12% vs 50%, respectively; *P* < .001).

**Conclusions:**

SUBA-itra was bioequivalent, well tolerated, and efficacious in treating endemic fungi, with a more favorable safety profile than c-itra.

**Clinical Trials Registration:**

NCT03572049.

## Teaser

Itraconazole is the mainstay therapy for endemic mycoses. Unfavorable pharmacokinetics lead to subtherapeutic levels and adverse effects. Super-bioavailability itraconazole is bioequivalent to conventional itraconazole, with similar clinical outcomes, but has fewer adverse events, possibly owing to lower drug-level variability.

Dimorphic fungi consist of numerous species. However, *Histoplasma*, *Coccidioides*, *Blastomyces*, and *Sporothrix* are the most common dimorphic fungi in North America, each occupying specific geographic ranges and ecological niches [1]. *Histoplasma* spp are common throughout the Mississippi, Ohio, and St Lawrence River valleys, the Caribbean, parts of Central and South America, Africa, and Asia [2–4]; *Coccidioides* within the southwestern United States, and portions of Central and South America [5]; *Blastomyces* within the Mississippi and Ohio River basins, St Lawrence Seaway, and several Canadian provinces, with sporadic cases in Africa, Central, and South America [6, 7]; and *Sporothrix* spp are found worldwide [8].

Current guidelines recommend itraconazole as the primary or an alternative first-line agent in treating non–life-threatening diseases caused by these endemic fungi or as step-down therapy in patients receiving amphotericin B for life-threatening infections [9–14]. However, the oral bioavailability of conventional itraconazole (c-itra) is variable and influenced by formulation, coingestion of food, and concurrently administered medications that affect gastric acidity and motility [15]. These factors affect itraconazole exposure and may reduce drug efficacy [16].

A new formulation, super-bioavailability (SUBA) itraconazole (SUBA-itra), has been developed and contains a solid dispersion of itraconazole in a pH-dependent polymeric matrix to enhance both dissolution and intestinal absorption [17]. Applying the SUBA technology to itraconazole has significantly increased oral bioavailability (173%) while reducing interpatient variability compared with the traditional oral formulation [17]. In addition, studies in healthy volunteers have shown that this novel formulation has little food or acid effect on bioavailability, a significant advance over c-itra [18, 19]. This study was designed to compare the efficacy and safety of SUBA-itra with those of c-itra in adult patients with endemic mycoses.

## METHODS

### Study Design

This was a prospective, multicenter, randomized, open-label, parallel-arm study involving patients with proven or probable endemic fungal infection to ascertain the safety, efficacy, and tolerability of oral SUBA-itra compared with c-itra (Clinicaltrials.gov NCT03572049); c-itra was chosen owing to a lower adverse effect profile compared with the liquid formulation.

### Patient Consent

Ethics committees or institutional review boards at participating sites approved the protocol and all amendments. Patients or their legally authorized representatives provided written informed consent. The trial was conducted at 10 centers in 2 countries, following current country and local regulations, the International Conference on Harmonization Good Clinical Practice, and the Declaration of Helsinki.

### Patient Disposition

Eligible patients aged ≥18 years with proven or probable infection with endemic mycosis (*Histoplasma*, *Coccidioides*, *Paracoccidioides*, *Blastomyces*, *Sporothrix*, or *Talaromyces marneffei* [formerly *Penicillium marneffei*]) according to current European Organisation for Research and Treatment of Cancer/Mycoses Study Group criteria [20] were enrolled. Key exclusion criteria were the use of alternative antifungal therapy (intravenous or oral) for >14 days, significant liver dysfunction (aspartate aminotransferase, alanine aminotransferase, alkaline phosphatase or total bilirubin levels >5 times the upper limit of normal), evidence of central nervous system infection, inability to take oral medications, a known history of congestive cardiac failure or ventricular dysfunction, and lactation or pregnancy. Patients with coccidioidomycosis who had previously received >14 days of fluconazole were included if they exhibited an inadequate response or were intolerant of therapy owing to adverse events (AEs) (patients required cessation of fluconazole for 7 days before enrollment).

### Randomization

**Figure 1.**
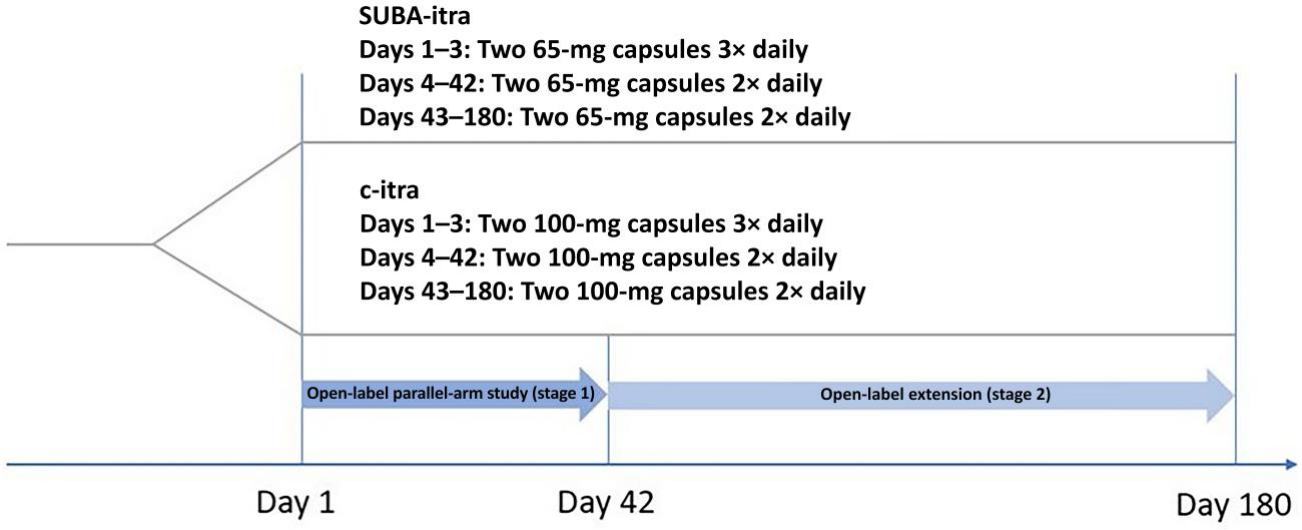
Flow diagram of study design for MSG-15. Patients were enrolled into the study and randomized to super-bioavailability itraconazole (SUBA-itra) or conventional itraconazole (c-itra), followed up for 42 days in an open-label parallel-arm study (stage 1), and, if clinically indicated, further enrolled into an open-label extension (stage 2), up to 180 days.

Patients were randomized (1:1), stratified for human immunodeficiency virus status, to c-itra or SUBA-itra. Patients randomized to SUBA-itra received 130 mg 3 times daily for 3 days, followed by 130 mg twice daily on days 4–42 (stage 1—open-label parallel-arm study). Subsequently, they received 130 mg twice daily on days 43–180 (stage 2—open-label extension) (Figure 1). Patients randomized to c-itra capsules received 200 mg thrice daily for 3 days, 200 mg twice daily on days 4–42 (stage 1), and 200 mg twice daily on days 43–180 (stage 2). Dose modification was permitted at the discretion of site investigators, with recommendations to make dose adjustments for itraconazole plus hydroxyitraconazole blood levels <500 ng/mL (increase dosing) or >5000 ng/mL or drug-related AEs, serious AEs, or dose-dependent drug toxicity (decrease dosing).

### Efficacy and Safety Assessments

All consented and randomized patients were included in the intent-to-treat population. Patients receiving any study medication were included in the safety population.

A 2-stage process was used to compare the efficacy, safety, tolerability, and pharmacokinetics (PK) of SUBA-itra versus c-itra. During stage 1 (days 1–42 of therapy), tolerability in both arms at day 42 and PK at day 14 were compared. In addition, efficacy, safety, and quality of life (QOL) at day 42 and PK at days 7 and 42 were compared during this stage. During stage 2 (days 43–180), efficacy, tolerability, safety, and QOL (including hospitalization and intensive care unit [ICU] status) were compared by patient group.

All consented and randomized patients were included in the intent-to-treat) population. Patients receiving either study medication were included in the safety population.

Outcomes were adjudicated by a data review committee of 3 independent experts who were not investigators in the trial. Clinical success was defined as the partial or complete resolution of attributable clinical symptoms, physical findings, and radiographic abnormalities attributed to infection. Failure was defined as the lack of improvement or worsening of attributable clinical signs, physical findings, or radiologic imaging. Mycologic outcomes were categorized as success (eradication or presumed eradication) or failure (persistence or presumed persistence).

The QOL evaluations were performed using the 12-Item Short Form Health Survey (SF-12; version 2)—a 12-question multipurpose survey instrument derived from the larger 36-Item Short Form Health Survey (SF-36) [21]. The 8 survey domains are physical functioning, role-physical, bodily pain, general health, vitality, social functioning, role-emotional, and mental health. The SF-12 (version 2) was used to determine the patient's overall well-being and health-related QOL by assessing physical and psychological status on days 1, 42, and 180. Safety was determined by recorded AEs, treatment-emergent AEs (TEAEs), all-cause mortality rate through the last known follow-up date, vital signs, laboratory testing, and electrocardiographic monitoring.

### Statistical Analysis

We used *t* and χ^2^ tests were used to compare the treatment groups. If it was determined that the data did not meet the assumptions of the *t* test, nonparametric statistical tests were used; Fisher's exact test was used as necessary. For nonparametric comparisons, we used the Kruskal-Wallis test. Differences were considered statistically significant at *P* ≤ .05 (2 sided). All analyses were performed using SAS software, version 9.4.

## RESULTS

### Study Conduct and Patient Disposition

Between 28 June 2018 and 15 January 2021, 88 patients with proven or probable endemic mycoses were enrolled (51 with histoplasmosis, 18 with blastomycosis, 13 with coccidioidomycosis, and 6 with sporotrichosis) (Supplementary Table 1). Treatment groups were well balanced and matched in demographics and baseline characteristics (Table 1). Of 88 patients, 68 (78%) completed the therapy. Specific infection did not affect the completion of treatment (Table 2).

**Table 1.** Baseline Characteristics (Intent-to-Treat Population) in 88 Patients With Endemic Mycoses Enrolled in Study Comparing Conventional and Super-Bioavailability Itraconazole, 2018–2021

	Patients, No. (%)		
Baseline Characteristic	SUBA-itra Group (n = 42)	c-itra Group (n = 46)	*P* Value
Age, years			
Mean (SD)	52.9 (18.2)	48.1 (17.4)	.22
Range	18–79	19–80	
Age <65 y	28 (67)	37 (80)	.15
Age ≥65 y	14 (33)	9 (20)	
Sex			
Male sex	26 (62)	26 (57)	.66
Race			
Asian	4 (10)	2 (0)	.03
Black	10 (24)	7 (15)	
White	27 (64)	32 (70)	
Other	1 (2)	7 (15)	
Ethnicity			
Hispanic/Latino	0 (0)	6 (13)	.056
Not Hispanic/Latino	41 (98)	41 (87)	
Not reported	1 (2)	0 (0)	
Diagnosis			
Blastomycosis	9 (21.4)	9 (19.6)	.18
Coccidioidomycosis	9 (21.4)	4 (8.7)	
Histoplasmosis	23 (54.8)	28 (60.9)	
Sporotrichosis	1 (2.4)	5 (10.9)	
Comorbid conditions			
HIV	2 (5)	2 (4)	>.99
Smoker status	23 (55)	25 (54)	
Chronic lung disease	3 (7)	5 (11)	.72
Autoimmune	7 (17)	10 (22)	.60
Solid organ transplant	5 (12)	7 (15)	.76
Diabetes mellitus	10 (24)	9 (20)	.80
Renal insufficiency	3 (7)	5 (11)	.72
Sarcoidosis	1 (2)	1 (2)	>.99
Stem cell transplant	1 (2)	1 (2)	>.99
Hepatic insufficiency	1 (2)	2 (4)	>.99
Corticosteroids	14 (33)	9 (20)	.15
Immunosuppressive therapy	14 (33)	15 (33)	>.99
SF-12 score, mean (SD)	38.8 (12.)	38.5 (11.0)	.95
BMI, mean (SD)	27.0 (6.4)	26.6 (6.2)	.78

Abbreviations: BMI, body mass index; c-itra, conventional itraconazole; HIV, human immunodeficiency virus; SD, standard deviation; SF−12, 12-Item Short Form Health Survey; SUBA-itra, super-bioavailability itraconazole.

^a^BMI calculated as calculated as weight in kilograms divided by height in meters squared.

**Table 2.** Completion of Therapy by Underlying Organism and Group Randomization in 88 Patients With Endemic Mycoses Enrolled in Study Comparing Conventional and Super-Bioavailability Itraconazole, 2018–2021

		Patients, No.	
Treatment Group	Mycosis	Therapy Completed Through Day 180	Withdrawal From Study
SUBA-itra	Blastomycosis	8	1
	Coccidioidomycosis	7	2
	Histoplasmosis	17	6
	Sporotrichosis	1	0
c-itra	Blastomycosis	6	3
	Coccidioidomycosis	3	1
	Histoplasmosis	23	5
	Sporotrichosis	3	2
Total	68	20	

Abbreviations: c-itra, conventional itraconazole; SUBA-itra, super-bioavailability itraconazole.

### Blastomycosis

Eighteen patients with blastomycosis were enrolled and randomized to SUBA-itra or c-itra (each n = 9). Eight patients had pulmonary, 5 had disseminated, 3 had cutaneous, and 1 each had osseous and combined osseous and pulmonary disease.

### Coccidioidomycosis

Thirteen patients with coccidioidomycosis were enrolled and randomized to SUBA-itra (n = 9) or c-itra (n = 4). Ten patients had pulmonary, 2 had disseminated disease, and 1 had osseous disease.

### Histoplasmosis

Fifty-one patients with histoplasmosis were enrolled and randomized to SUBA-itra (23) and c-itra (28). The most common site of infection was disseminated (25), followed by pulmonary (24), bone (1), and intestinal disease (1).

### Sporotrichosis

Six patients with sporotrichosis were enrolled and randomized to SUBA-itra (n = 1) and c-itra (n = 5). The most common site of infection was bone (n = 4), followed by skin (n = 1) and disseminated (n = 1) disease.

### Postrandomization Hospitalization

After randomization, patients receiving SUBA-itra had shorter hospitalization durations in stages 1 and 2 than those receiving c-itra, although these differences were not significant. The mean duration of hospitalization during days 1–42 was 4.56 days for the SUBA-itra group versus 5.76 days for c-itra (*P* = .78), with 0.03 and 0.10 of those days being in the ICU (*P* = .98), respectively. During days 43–180, the hospital stays were much shorter for the SUBA-itra and c-itra groups at 0.55 versus 0.77 days (*P* = .67), respectively, with no ICU admissions. With the SF-12 tool, there were no differences between the 2 arms in QOL regarding routine daily activities, sense of well-being, or overall emotional health (Supplementary Tables 2 and 3).

### Mortality and Clinical Response

Only 1 death occurred in the SUBA-itra arm on day 13. This death was not thought to be related to the underlying endemic mycosis or the SUBA-itra therapy.

The clinical responses for SUBA-itra and c-itra were similar at day 42 (49% vs 67%, respectively) and day 180 (60% vs 65%) (*P* = .13). Mycologic responses were also similar on day 42 (45% vs 48%, respectively; *P* = .83) and day 180 (40% vs 48%; *P* = .53) (Table 3).

**Table 3.** Clinical and Mycologic Outcomes at Days 42 and 180 in 88 Patients With Endemic Mycoses Enrolled in Study Comparing Conventional and Super-Bioavailability Itraconazole, 2018–2021

Outcome	Patients, No. (%)			
	Day 42		Day 180	
	Clinical	Mycologic	Clinical	Mycologic
SUBA-itra group (n = 42)				
Success	29 (69)	19 (45)	25 (60)	17 (40)
Failure	8 (19)	9 (21)	6 (14)	7 (17)
Unevaluable	5 (12)	14 (33)	11 (26)	18 (43)
c-itra group (n = 46)				
Success	31 (67)	22 (48)	30 (65)	22 (48)
Failure	7 (15)	14 (30)	5 (11)	9 (20)
Unevaluable	8 (17)	10 (22)	11 (24)	15 (33)

Abbreviations: c-itra, conventional itraconazole; SUBA-itra, SUBA itraconazole.

The clinical responses for SUBA-itra and c-itra were 78% and 67%, respectively, at day 42 (*P* = .60) and 100% and 75% at day 180 (*P* = .19) in patients with blastomycosis, 63% and 100% at day 42 (*P* = .30) and 63% and 100% at day 180 (*P* = .45) in patients with coccidioidomycosis, 89% and 80% at day 42 (*P* = .40) and 81% and 83% at day 180 (0.87) in patients with histoplasmosis, and 0% and 50% at day 42 (*P* = .36) and 100% and 10% at day 180 in patients with sporotrichosis.

### Pharmacokinetics

**Figure 2.**
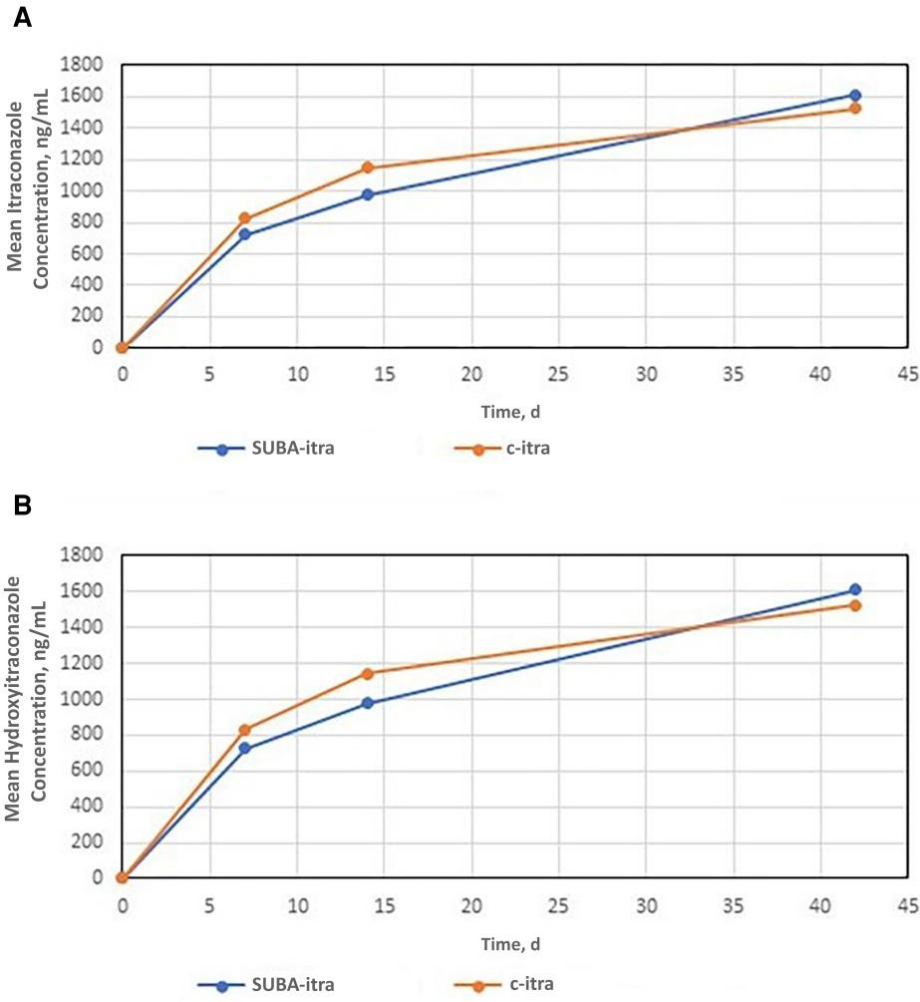
Mean concentrations of itraconazole and hydroxyitraconazole in patients infected by dimorphic fungi. Super-bioavailability itraconazole (SUBA-itra) demonstrated bioequivalence with conventional itraconazole (c-itra) with respect to both itraconazole and hydroxyitraconazole levels on days 0–42.

There was no difference between the area under the curve for SUBA-itra and c-itra in either the itraconazole component (1963 vs 1897, respectively; *P* = .80 d*ng/ml) or the hydroxyitraconazole component (1015 vs 1058; *P* = .77 d*ng/ml) (Table 4 and Figure 2). Less variability in drug levels was observed on days 7 (*P* = .03) and 14 (*P* = .06) in the SUBA-itra group. Once dose adjustments were made, drug-level variability on day 42 was comparable between the 2 groups (*P* = .41).

**Table 4.** Area Under the Curve Values for (A) Itraconazole and (B) Hydroxyitraconazole

Measurement	AUC (d*ng/ml)		*P* Value
	SUBA-itra Group	c-itra Group	
Hydroxyitraconazole	1963	1897	.80
Itraconazole	1015	1058	.77

Abbreviations: AUC, area under the curve; c-itra, conventional itraconazole; SUBA-itra, SUBA itraconazole.

Overall, 17 patients (19%) required dose adjustments, with 9 of 42 (21%) in the SUBA-itra arm and 8 of 46 (17%) in the c-itra arm. In the SUBA-itra arm, 2 patients required 2 changes, and 1 required 3. The 2 patients who were switched twice were switched early in the therapy course to low PK levels and subsequently returned to the original dose with appropriate levels. In the c-itra arm, 1 patient required 2 dosing changes, and 1 required 4. Unlike in the c-itra arm, no patients in the SUBA-itra arm needed their dosing to increase above the recommended 130 mg twice daily.

### Tolerability and Adverse Events

Tolerability on days 14 (*P* = .70), 28 (*P* = .05), 42 (*P* = .20), 84 (*P* = .29), and 180 (*P* = .19) was comparable in both groups (Supplementary Table 4). The percentage of patients who experienced ≥1 AE) was 74% in the SUBA-itra and 87% in the c-itra group (*P* = .18). The most common TEAEs in the SUBA-itra and c-itra groups were cardiovascular (33% vs 41%, respectively) and gastrointestinal (7% vs 20%). All other TEAEs occurred in <1% of patients in both groups (Table 5).

**Table 5.** Adverse Events in 88 Patients With Endemic Mycoses Enrolled in Study Comparing Conventional and Super-Bioavailability Itraconazole, 2018–2021

System	AEs in SUBA-itra Group, No.			AEs in c-itra Group, No.		
	Total AEs	TEAEs	SAEs	Total AEs	TEAEs	SAEs
Respiratory	8	0	1	18	1	1
Reproductive	1	0	0	2	0	1
Renal	2	0	0	8	0	0
Psychiatric	0	0	0	2	0	0
Ophthalmologic	3	0	0	3	0	0
Neurologic	15	1	0	15	0	1
Musculoskeletal	12	0	0	12	1	1
Metabolic	1	0	1	4	0	0
Integumentary	12	1	1	15	1	0
Infectious	5	0	0	10	0	3
Hepatobiliary	3	0	0	9	1	2
Hematologic	5	1	0	5	1	0
General	27	1	0	16	0	2
Gastrointestinal	23	3	0	26	9	8
Cardiovascular	25	14	2	34	19	4
Total	142	21	5	179	33	23

Abbreviations: AEs, adverse events; c-itra, conventional itraconazole; SAEs, serious AEs; SUBA-itra, SUBA itraconazole; TEAEs, treatment-emergent AEs.

Serious TEAEs were reported in 12% in the SUBA-itra group (2 cardiovascular, 1 integumentary, 1 metabolic, and 1 respiratory) and 50% in the c-itra group (4 cardiovascular, 8 gastrointestinal, 2 general, 2 hepatobiliary, 3 infectious, and 1 each of musculoskeletal, neurologic, reproductive, and respiratory) (*P* < .001).

Two patients were withdrawn from the c-itra group for lack of efficacy, but none were withdrawn from the SUBA-itra group for the same reason. Other reasons for withdrawal were consent withdrawn/loss to follow-up (n = 3), AE (3), death (n = 1), and completion of therapy before day 180 (n = 2) in the SUBA-itra arm, and consent withdrawn/loss to follow-up (n = 4), AE (n = 3), and other (n = 4) in the c-itra arm.

## DISCUSSION

In this prospective, multicenter, randomized, open-label, parallel-arm study, we compared the safety, efficacy, and PK of SUBA-itra with those of c-itra in the treatment of 4 different endemic mycoses. Our findings show that SUBA-itra achieves similar levels as c-itra with a dose approximately one-third lower. In our study, patients receiving SUBA-itra had less variability in their itraconazole blood levels. They experienced fewer AEs and TEAEs than patients in the c-itra arm.

Treating endemic mycoses can be difficult for clinicians and patients. Currently available agents are limited by toxicity, poor oral bioavailability [22], or resistance. Fluconazole is widely available yet has limited activity against several endemic mycoses, including histoplasmosis [23]. In others, such as coccidioidomycosis, treatment at high doses (>800 mg/d) is routinely required [11], limiting patient tolerability [24]. Voriconazole has been associated with cutaneous cancer, photosensitivity [25], fluorosis [26, 27], hepatotoxicity, and PK variability between patients, has limited activity against *Sporothrix* spp, and appears to have decreased activity against *Histoplasma* [14]. Posaconazole solution similarly exhibits dietary requirements and absorption concerns that limit patient compliance and bioavailability [28] and may cause hypertension in some patients [29]. Experience with posaconazole tablets and isavuconazole remains limited for these infections [30, 31].

Despite its variable absorption, food/acidity requirements, and limited tolerability, c-itra remains the standard of care treatment for most endemic mycoses. The advent of SUBA-itra may circumvent some of these issues. Specifically, SUBA-itra releases drug in the duodenum—unlike c-itra, which is released in the stomach—improving relative bioavailability by 173%, with a 21% decrease in intrapatient variability compared with c-itra capsules [17]. Only moderate food effects have been observed with SUBA-itra, with C_trough_ levels in the fed and fasted state within 10% of each other [3]. When a single dose of SUBA-itra was coadministered with a proton pump inhibitor after establishment of a steady state, plasma exposure was increased [18], removing a significant barrier to treatment in many patients. These are key attributes that may improve serum drug levels and treatment efficacy.

In our study, no patient required an increase in SUBA-itra dose above 130 mg twice a day, the recommended dose for SUBA-itra, likely owing to better absorption. In addition, no patient receiving SUBA-itra had to withdraw from the study owing to lack of efficacy, which may have been related to more favorable PK.

In our study, no mycoses-related deaths occurred. This is unsurprising, as the mortality rate in patients with mild-to-moderate disease requiring only azole therapy is negligible [3, 14, 32]. In patients with severe disease, deaths tend to occur early, during the induction phase, while patients are being treated with polyenes [33]. As such, we would expect this cohort's mortality rate to be low.

It is of interest that fewer AEs and severe AEs were observed in the SUBA-itra group. Cardiovascular and gastrointestinal/hepatobiliary events were the most common. These observations may be due to improved PK and lower gastrointestinal exposure [19, 34, 35]. While the area under the curve was similar between the 2 groups, especially once the dosing adjustments were made, the variability was significantly higher in the c-itra arm. This suggests that while the means and medians were similar, the standard deviations were different, as more patients in the c-itra arm had undesirably low or high levels of itraconazole and hydroxyitraconazole in their blood. We suspect that these patients drove the difference in the tolerability and AEs.

In addition, SUBA-itra is absorbed in the duodenum, and a significantly higher portion of the orally administered drug is absorbed [17]. As a result, the rest of the small intestine experienced considerably lower concentrations of intraluminal itraconazole, decreasing the drug exposure of the intestinal mucosa. This is likely an important reason for reduced gastrointestinal AEs. These AEs in our study were comparable in magnitude and type to those in prior itraconazole studies (25%–57%). Treatment discontinuation was required in 1%–2%, similar to our study [36–38].

The lower rates of adverse effects were not measurable in our QOL instrument, suggesting that the decline in QOL that patients with endemic mycoses experience is related more to the disease, and the lessening of AEs did not translate to an improvement significant enough to be measured by our instrument.

In the present study, the efficacy of SUBA-itra was comparable to that of c-itra for treating endemic mycoses. These findings are consistent with prior evaluations of c-itra in treating endemic fungal infections [32, 36, 37, 39–41]. These previous studies evaluated c-itra at 100–600 mg/d. In blastomycosis, early studies using 200–400 mg/d found that c-itra was effective in 90% of patients [37]. In the treatment of coccidioidomycosis, c-itra (300–400 mg/d) has been evaluated in nonmeningeal and meningeal disease, with favorable responses in both populations [32, 42]. In the treatment of histoplasmosis, studies using 200–400 mg/d have found efficacy rates of 81%, with failures typically seen in those with chronic cavitary pulmonary disease [37].

Patients with sporotrichosis received doses between 100 and 400 mg/d. Those with lymphangitis and cutaneous forms of disease had efficacy rates exceeding 94%, despite doses as low as 100 mg/d [40, 41]. Those with articular and osseous disease typically require higher doses (100–600 mg/d) and exhibit lower response rates and a higher relapse rate, reflecting the difficulty in treating these forms of disease [32, 39]. Our results were similar, with 11% and 14% of patients experiencing clinical failure at day 180 in the SUBA-itra and c-itra groups, respectively. This suggests that in addition to previously well-defined bioequivalence, SUBA-itra achieves clinical outcomes similar to those achieved with c-itra.

The exclusion of patients with meningitis, paracoccidioidomycosis, and talaromycosis limits this study. While it is the largest prospective study of endemic mycoses in more than 20 years, the size of each pathogens group significantly limits its interpretability.

In summary, SUBA-itra, compared with c-itra, effectively treated selected dimorphic fungal infections and appeared to have less PK variability and fewer AEs. The observed outcomes suggest it that it is a welcome addition to the antifungal armamentarium.

## Supplementary Material

ofae010_Supplementary_Data

## Supplementary Data

Supplementary materials are available at *Open Forum Infectious Diseases* online. Consisting of data provided by the authors to benefit the reader, the posted materials are not copyedited and are the sole responsibility of the authors, so questions or comments should be addressed to the corresponding author.
