# Supplementary material for: MSG-15: Super-Bioavailability Itraconazole Versus Conventional Itraconazole in the Treatment of Endemic Mycoses—A Multicenter, Open-Label, Randomized Comparative Trial
Source: Open Forum Infect Dis. 2024 Jan 29;11(3):ofae010. doi: 10.1093/ofid/ofae010 (PMC10911225; doi:10.1093/ofid/ofae010)
Supplement: ofae010_Supplementary_Data [file ofae010_supplementary_data.docx]

**Supplemental Table 1: Study Enrollment by Organism and Randomization**

| Mycosis | SUBA-itra (%) | C-itra (%) | Total |
| --- | --- | --- | --- |
| Blastomycosis | 9 (50) | 9 (50) | 18 |
| Coccidioidomycosis | 9 (69) | 4 (31) | 13 |
| Histoplasmosis | 23 (45) | 28 (55) | 51 |
| Sporotrichosis | 1 (17) | 5 (83) | 6 |
| Total | 42 | 46 | 88 |

**Table 2.** **SF-12 Physical Component Summary Scores**

| Drug | Timepoint | N | Mean | Median |
| --- | --- | --- | --- | --- |
| SUBA-itra | Baseline | 40 | 38.85 | 38.71 |
|  | Day 42 | 38 | 43.81 | 45.60 |
|  | Day 180 | 31 | 45.98 | 48.47 |
| c-itra | Baseline | 42 | 38.55 | 39.96 |
|  | Day 42 | 41 | 43.47 | 44.05 |
|  | Day 180 | 31 | 46.70 | 47.57 |

**Table 3. SF-12 Mental Component Summary Scores**

| Group | Timepoint | N | Mean | Median |
| --- | --- | --- | --- | --- |
| SUBA-itra | Baseline | 40 | 47.96 | 51.81 |
|  | Day 42 | 38 | 53.49 | 56.45 |
|  | Day 180 | 31 | 50.49 | 52.83 |
| c-itra | Baseline | 42 | 48.90 | 51.46 |
|  | Day 42 | 41 | 54.80 | 56.53 |
|  | Day 180 | 31 | 57.05 | 58.74 |

Table 4. Tolerability of therapy by drug over course of study.

| Description | Study Treatment | Day 14  (n) | Day 28  (n) | Day 42  (n) | Day 84  (n) | Day 180  (n) |
| --- | --- | --- | --- | --- | --- | --- |
| *Patient is tolerating the therapy very well* | SUBA-Itra | 33 | 35 | 33 | 32 | 25 |
|  | C-itra | 35 | 33 | 37 | 30 | 26 |
| *Patient is tolerating the therapy well but occasionally feels badly* | Suba-itra | 4 | 2 | 3 | 3 | 4 |
|  | C-itra | 7 | 4 | 0 | 2 | 2 |
| *Patient is tolerating the therapy well but feels badly about half the time* | Suba-Itra | 3 | 3 | 3 | 3 | 4 |
|  | C- itra | 1 | 0 | 1 | 0 | 0 |
| *Patient is tolerating the therapy but often feels badly* | Suba-itra | 0 | 0 | 1 | 0 | 0 |
|  | C- itra | 1 | 4 | 3 | 1 | 1 |
| *Patient is not tolerating the therapy and is no longer able to continue the medication.* | Suba-itra | 1 | 0 | 0 | 0 | 3 |
|  | C-itra | 1 | 0 | 1 | 1 | 1 |
